# Public opinion towards global distribution of COVID-19 vaccines - Data from Germany and the United States

**DOI:** 10.1038/s41597-022-01700-z

**Published:** 2022-09-23

**Authors:** Matthias Klumpp, Ida G. Monfared, Sebastian Vollmer

**Affiliations:** 1 Georg-August-University of Göttingen, Department of Business Administration, Chair for Production and Logistics, Platz der Göttinger Sieben 3, 37073 Göttingen, Germany; 2 Georg-August-University of Göttingen, Department of Economics, Centre for Modern Indian Studies, Waldweg 26, 37073 Göttingen, Germany

**Keywords:** Health care, Disease prevention

## Abstract

This study gathered evidence from Germany and the United States on public opinion towards fair distribution of COVID-19 vaccines across the world. Analytical Hierarchy Process and discrete choice experiments were used for this purpose. The sample is nationally representative of adults (aged 18 and above) for both countries using quotas on age, gender, education, state, and COVID-19 vaccination rates at the time of the fieldwork (25 May 2021 to 26 June 2021). Overall 1,003 responses in Germany and 1,000 in the United States were collected.

## Abstract (summary)

**Table:** 

Measurement(s)	Public opinion
Technology Type(s)	Questionnaire

## Background & Summary

Supply of COVID-19 vaccines remains uneven across the world. While high-income countries could ensure that they would have access to more than enough supplies, many low- and middle-income countries have not yet vaccinated their vulnerable population with a single dose. The present study investigates the public opinion towards fair global distribution of the vaccines in two high-income countries, Germany and the United States, in which the first two mRNA vaccines were developed.

Following theories of distributive justice^1,2^, this study^3^ proposes four main principles to be weighted by the public to identify these grounds relative importance: that vaccines should be distributed according to better outcomes (utilitarian), equally (egalitarian), based on merits (R&D), or based on free market rules^4,5^. While these were the main interpretations used in our study, other interpretations and uses of the survey items are possible, for instance using the concept of prioritarianism instead of utilitarianism.

There are a small number of other studies which looked at the public opinion towards the global distribution of vaccines that were mostly conducted at earlier stages of the pandemic. E.g. Guidray *et al*. carried out a survey in the United States in July 2020^6^ and Clarke *et al*. surveyed the public opinion in Australia, Canada, France, Italy, Spain, the UK and the USA in Nov.-Dec. 2020^7^ where at these points in time, the vaccination campaigns were mainly limited to the most vulnerable groups. Later evidence were also gathered e.g. in the UK in late August 2021^8^ where results indicated public support for donation of the vaccines over booster shots.

Our data captured the public opinion at a critical point in time when the adult population themselves were waiting to receive their first dose of the vaccine and a gradual rollout of the appointments was in progress. At this time, the public mindset might have been different from any other point, particularly when it came to self-interest bias.

## Methods

The survey instrument consisted of four main sections. In the first module, with the help of the Analytical Hierarchy Process (AHP) method, interviewees were asked regarding a fair distribution of COVID-19 vaccines across the world which motivation and reasoning they judged to be most important. AHP was used to allow for a relatively unbiased rational choice response in using the pairwise comparisons of the method. Specific items were “equal access for all”, “medical urgency”, “free market rules apply”, and “production contribution”, standing for egalitarian, utilitarian, libertarian, and motivations. The items are asked for and compared in the AHP as pairwise comparisons, so for example judging between “equal access for all” and “production contribution” as a direct evaluation on a 17-scale system provided by AHP. The second module was a discrete choice experiment, where respondents had to divide a hypothetical 100 million doses of vaccines between the two hypothetical countries A and B. These two countries resemble a high- and low-income country, e.g. country B had 300 million inhabitants, 3,000 COVID-19 deaths per day, and ordered 100 million vaccine doses while country A had 100 million inhabitants, 200 COVID-19 deaths per day, and had ordered 1,000 million doses of vaccines. This experiment was first carried out under the veil of ignorance with no further information. In a second run, respondents were asked to think of a vulnerable family member being on the waiting list in country A with the information that this person’s place on the weighting was equal to the amount allocated to country A in the previous scenario plus 10 million. In the third run, respondents were asked to play the same allocation game but this time, consider themselves to be on the waiting list in a place where they needed to add 30 million additional doses to the amount given to country A to receive the vaccine themselves. These changes of scenarios added an element of self-interest at a time when many respondents were still waiting for their first dose of the vaccine in June 2021. In a third module, respondents were asked for their level of agreement (on a 7-point Likert scale with 1 = strongly disagree to 7 = strongly agree) to wait for their own vaccine for 3 months so that people in countries with specified characteristics could be vaccinated earlier. These characteristics – which were individually presented – were “larger population size”, “higher number of COVID-19 deaths”, “lower number of intensive care hospital beds”, “higher number of vaccine pre-orders”, “higher annual income per head”, “investment in research and development of the vaccines”, and “production capacity for vaccines”. The fourth module was a second AHP asking respondents to rate the importance of seven detailed criteria for vaccine distribution that where “number of inhabitants”, “number of daily COVID-19 deaths”, “number of intensive care unit (ICU) beds per 100 k population”, “number of vaccines pre-ordered”, “annual income per head (GDP)”, “investment in vaccine research and development (R&D)”, and “vaccine production capacity”.

To control for potential biases, respondents were asked whether or not they have already been vaccinated, to what extend COVID-19 had a negative impact on their lives (7-point Likert scale, 1 = strongly disagree to 7 = strongly agree), and also regarding their tendency for social desirability bias using the short 5-item version of the Marlowe-Crowne scale (SD5). The survey instrument was trialled before implementation and data was monitored for quality throughout the duration of the fieldwork.

A sample size of 1,000 was implemented for each country surveyed, following the method used by Gallup for conducting national polls. Using quotas, the sample was drawn to be representative of the adult population (aged 18 and above) in Germany and the United States according to age, gender, education, state and current vaccination rates at time of the fieldwork, from 25 May 2021 to 26 June 2021. Participants were recruited by a professional survey agency (INFO located in Berlin). To ensure population representativeness, in particular for older age groups in Germany, respondents were initially contacted via phone calls. Then, all participants were invited to complete the survey online. Overall, 1,003 responses in Germany and 1,000 in the United States were collected. The study with reviewed and approved by the Ethics Committee of the University of Göttingen prior to data collection.

## Data Records

Data for this study is accessible via the University of Goettingen data depository platform, Göttingen Research Online^9^. The data folder comprises of three files: the data for Germany (Die globale Verteilung des COVID-19 Impfstoffes 28.06.2021 Datensatz), the data for the US (Global Distribution of COVID-19 Vaccines 28.06.2021 Dataset), and the survey questionnaire in English (Public attitude towards fairness in global distribution of COVID-19 vaccines_EXT_EN). The data files are in MS Excel format. Each data file has three tabs: 1) variable label, 2) code book, and 3) dataset. The first column in “variable label” tab shows the “variable name” and the second column is the “variable label”. Each “variable name” corresponds with its associated data column in the “dataset” tab. The “codebook” tab presents the response option categories and their codes in details. These datasets also include weights that have to be used in the analysis to ensure that samples are representative of each country’s population. The data is in its raw format and has not been manipulated. It should be noted that for the AHP modules, in order to extract the relative weighting of various criteria, an AHP calculator will be needed. There are various options for this purpose of which some are available for free^10^ and some are commercially accessible.

## Technical Validation

We monitored the data throughout the process and ensured its validity through various means. In the module respondents were asked to divide 100 Mill doses of vaccines between the two countries, each step was followed by a free-text question asking the respondents to elaborate on their line of thoughts and the reason behind their decision. These free-text comments (that are included in the present dataset) allowed to cross-reference the way in which the division was made with the respondents’ line of thoughts. For the AHP data, it is possible as part of the analysis to calculate the internal consistency for each respondent. Given that some questions carry ethical burden, the study also includes a measure to capture individual’s bias towards social desirability^11^. Moreover, the survey includes a question asking respondents to rate the extent to which their lives have been affected by the pandemic that can be used as another control measure for potential bias. We have conducted multiple pilots to ensure the accuracy of the questionnaire instrument and its clarity to the respondents. A number of pilots were conducted prior to the launch of the survey, also the first week of the fieldwork was treated as pilot to smooth out any issues.

### Ethics committee approval

This study received ethical approval from the ethics committee at the University of Göttingen, Germany.

## Data Availability

No custom code was used to generate this study.
